# Douching practices among women in the Bolgatanga municipality of the upper east region of Ghana

**DOI:** 10.1186/s12905-019-0720-7

**Published:** 2019-02-08

**Authors:** Florence Assibi Ziba, Vida Nyagre Yakong, Rockson Asaan Asore, Keville Frederickson, Michele Flynch

**Affiliations:** 1 Department of Nursing, University for Development Studies, Tamale, Ghana; 2 Department of Midwifery, University for Development Studies, Tamale, Ghana; 3 Bongo District Hospital, Ghana Health Service, Bongo, Ghana; 4 Lienhard School of Nursing, Pace University, Pleasantville, New York City, USA

**Keywords:** Douching practices, Knowledge, Women, Vaginal hygiene, Bolgatanga Municipality

## Abstract

**Background:**

Vaginal douching is a common practice among women all over the world. Women douche for various reasons, despite evidence indicating the harmful health effects of the practice. There is lack of data on the practice in Ghana but health behaviors like vaginal douching may be influenced by differences in culture and geography. Therefore, this study sought to assess prevalence and predictors of vaginal douching practices among women of the Bolgatanga Municipality of Ghana.

**Methods:**

This cross-sectional study was conducted among 200 women from January to March 2016.

**Results:**

Sixty-seven percent of the women practiced vaginal douching, from which a similar proportion did it daily. Over two-thirds (67.7%) of the women used water for douching. The reasons for douching were cleansing the vagina (67.7%), therapeutic effects (12.8%) and tightening of the vaginal muscles (19.5%). The majority (87.2%) of women who douched were unaware of the harmful health effects associated with the practice. The educational level of the women (*p* = 0.025) and having knowledge of the dangers associated with douching (*p* <  0.001) were then significantly associated with douching practices.

**Conclusion:**

Vaginal douching is commonly practiced by women in the Bolgatanga Municipality. Most of these women douche because they did not know that there are health problems associated with the practice. Health education on the issues of women health is very vital for the improvement of women’s health.

## Background

Vagina douching (VD) is the process of rinsing the vagina by introducing different kinds of fluids for the purpose of hygiene or therapeutic or contraception [1, 2]. This is a common practice among women all over the world dating back to many centuries with a prevalence of 25% among more developed countries such as Canada and the US to over 90% in less developed countries such as Zambia and Ghana [3, 4]. The prevalence however, varies from region to region and is influenced by certain socioeconomic factors such as race and level of education. It is believed that the practice is more common among women of the black race and those with low educational status [5, 6]. Many products are used for the purpose of douching [7]. In Sub-Saharan Africa, products such as herbs, chemicals, powder and papers are among substances inserted into the vagina [8]. Most of these products are found to be hazardous to women’s health by causing irritation to the vaginal mucosa [6].

Water is the most commonly used product for the practice of douching among women in Ghana [9]. There are few reports that focus on douching practices using water leading to specific negative outcomes. A qualitative study examined douching practices among Turkish women reported a connection between douching with water and genital and vaginal infections [10]. A case study reported the presence of leaches in an Ethiopian woman who used local water to douche [11]. Other complications related to the physical effects of the pressure created by douching, include ectopic pregnancy, preterm labor, low birth weight and infertility [12–16]. Despite these effects, women are motivated by certain reasons to continue the practice of vaginal douching. Some of these motivating factors include, sexual pleasure, the desire to tighten the vagina muscles, individual perception, belief and religion [6, 9, 17] . Data regarding douching practices and the awareness of its dangers by women in Ghana is still lacking. In the search for literature, only one study was found which was done in Accra, Greater Accra Region of Ghana on this topic.

This study was conducted in the Bolgatanga Municipality of Upper East Region of Ghana to estimate the prevalence and assess predictors of vaginal douching practices among women in the Municipality.

## Methods

### Setting

Ghana is a Sub-Saharan African country located in West of Africa. It shares boundaries with Togo to the east, Burkina Faso to the north, Cote d’Ivoire to the west and the Gulf of Guinea to the south. It is divided into ten [10] administrative regions and 216 districts.

The Upper East Region is the second smallest and poorest region in Ghana. The Bolgatanga Municipality is the largest urban centre among the 13 Districts and Municipalities of the Upper East Region of Ghana and also the regional capital. The main occupation of the people is agriculture and its related works which employ about 60% of the population.

According to the 2010 Population and Housing Census, the Municipality accounts for 12.6% of the population of the Upper East Region with a total population of 131,550. Females form 52.3% of this population and about 50.2% of the population are rural dwellers. The population of females aged 15–59 years in the Municipality is 38,917 [18].

Gurunui is the main local dialect spoken in the Municipality. It is located approximately 779 km north of Accra, Ghana’s capital city.

### Study design and sampling

A cross sectional study design was used. The women were selected using convenient sampling method. The sample size was estimated using the prevalence rates of vaginal douching from previous studies [19]. Hence, the formula $\mathrm{n}=\frac{{\mathrm{z}}^2\mathrm{P}\ \mathrm{q}}{\mathrm{d}2}$ was used to calculate the sample size [20].

n = Sample size

z = Standard normal deviation = 1.96 at 95% confidence level

P = Prevalence rate = 81.9%

q = 1-*P* = 1–0.819 = 0.181

d = Error margin = 5%

$$ {\displaystyle \begin{array}{c}\mathrm{n}=\frac{(1.96)^2{0.819}^{\ast }0.181}{(0.05)2}\\ {}=\frac{3.841^{\ast }0.148}{0.0025}\end{array}} $$

$$ \mathrm{n}=227.4 $$

The inclusion criteria for this study was all females aged 15–59 years, who resided in the Bolgatanga Municipality and understood the English Language or the Guruni local dialect. The women were contacted at their various homes and work places by a research assistant. The study was explained to them and those willing to participate were allowed to do so.

### Study design and data collection methods

Data was collected using a structured questionnaire. The questionnaire was made up of both closed and opened ended questions that assessed demographic data, knowledge level and douching practices. Copies of the questionnaires were given to participants who could read and understand the English Language. Subjects were then asked to complete the questionnaire at their own convenience and return to the researchers. For the non-English speakers, the questionnaire was translated into the local dialect and administered to those subjects on the spot.

### Statistical analysis

Data was analyzed using IBM Statistical Package for Social Sciences (SPSS 23.0). Descriptive statistics of frequencies and percentages were used to describe the data. Cross tabulations were used to determine associations among categorical variables using chi-square test. A *p*-value of less than 0.05 was considered significant.

### Ethical consideration

The study was granted ethical approval by the Research and Ethical Committee of the School of Allied Health Sciences of the University for Development Studies, Tamale, Ghana. Voluntary verbal informed consent was obtained from each study participant. Participants were told they could decide to withdraw from the study anytime without any consequences. The purpose of the study and the assurance of confidentiality of information were explained to participants. They were also told they could decide not to answer any question they felt uncomfortable with.

## Results

A total of 250 copies of a questionnaire were administered to the participants. However, 236 copies of them were returned in which 36 were incomplete resulting in a response rate of 80%.

Table 1 presents the demographic data of the participants. The majority (83%) of the participants were within the ages of 15–30 years, 66% were single, 71.5% were Christians, Muslims constituted 27% whiles over 90% had at least a minimum (Junior High School) level of formal education.

**Table 1:** Socio-demographic characteristics of the women in the total sample

Variables	Frequency (%)
Age	
15–20	80(40)
21–30	86(43)
31–40	26(13)
41–50	7(3.5)
> 51	1(0.5)
Marital status	
Single	132(66)
Married	65(32.5)
Divorced	3 (1.5)
Religion	
Christianity	143(71.5)
Islam	54(27)
Educational level	
No formal education	11(5.5)
Primary	9(4.5)
Junior High	12(6)
Senior High	82(41)
Tertiary	86(43)

Table 2 presents data on the douching practices of the participants. Over 60% of them indicated they douche with 67% of them reporting doing so daily Most of those who douche, (67.7%) used water as the product for douching, 19.5% use lemon juices whiles the rest use antiseptics (9.8%) and or vinegar (3.0%). The reasons for douching were mainly for cleansing the vagina (67.7%), tightening the vaginal muscles (19.5%) and or for therapeutic reasons (12.8%). The desire to please their spouse sexually influenced most (59.4%) of the women to douche, followed by tradition (21.1%) and religion (19.5%).

**Table 2:** Douching practices

Variables	Frequency (%)
Douching	
Yes	133(66.5)
No	67(33.5)
Frequency of douching (*n* = 133)	
Daily	89(67.0)
Weekly	18(13.5)
When necessary	26(19.5)
Products used for douching	
Water	90(67.7)
Lemon Juice	26(19.5)
Antiseptics	13(9.8)
Vinegar	4(3.0)
Factors that determine the product used	
Purpose	44(33.1)
Availability	55(41.4)
Reasons for douching	
Therapeutic	17(12.8)
Cleansing of the vaginal	90(67.7)
Tighten of the vaginal muscles	26(19.5)

Friends (62.4%) were the primary source of information for those who had ever heard of douching, parents (17.3%), media (9.8%), self-experimentation (9.0%) and partners were the other sources of information for participants who practiced douching. The majority (87.2%) of those who douched stated that they did not know of any dangers associated with douching as indicated in Table 3 below.

**Table 3:** Knowledge of participants about douching

Variables	Frequency (%)
Source of information about douching practices	
Friends	83(62.4)
Parents	23(17.3)
Media	13(9.8)
Partners	2 (1.5)
Self-experimentations	12(9.0)
Knowledge on dangers associated with douching	
Yes	17(12.8)
No	116(87.2)

Douching practice was less among participants who had tertiary level of education (56.7%) and those who also knew the dangers associated with douching (92.5%). Age and marital status of the participants were not associated with douching practices among these two categories of women. However, level of education and not knowing the dangers associated with douching practice were strongly associated with the practice with *P*-values of 0.047 and <  0.001 respectively as indicated in Table 4 below.

**Table 4:** Factors associated with douching practice

Variables	Douching practices		*p*-value
	Yes	No	
Age			
15–20	55(41.4%)	25(37.3%)	0.891
21–30	56(42.1%)	30(44.8%)	
31–40	16(12.0%)	10(14.9%)	
41–50	5(3.8%)	2(3.0%)	
Above 50	1(0.8%)	0(0%)	
Marital status			
Single	84(63.2%)	48(71.6%)	0.232
Married	48(36.1%)	17(25.4%)	
Divorced	1(0.8%)	1(1.5%)	
Widowed	0(0%)	1(1.5%)	
Educational status			
JHS and below	26(19.5%)	6(9.0%)	0.047
SHS	59(44.4%)	23(34.3%)	
Tertiary	48(36.1%)	38(56.7%)	
Knowing the dangers of douching			
Yes	68(51.1%)	62(92.5%)	< 0.001
No	26(19.5%)	0(0.0%)	
Don’t know	39(29.3%)	5(7.5%)	

A Chi – Square Test. *P*-value of 0.05 was considered significant

As indicated in Table 5 below, participants with higher level of education were more aware of the dangers associated with douching compared to their counterparts.

**Table 5:** Educational level*Knowing dangers associated with douching

Variables	Know dangers associated with douching			
Educational status	Yes	No	Don’t know	*P*-value
Primary	10(7.7%)	5(19.2%)	5(11.4%)	0.035
Secondary	53(40.8%)	12(46.2%)	29(66.0%)	
Tertiary	67(51.5%)	9(34.6%)	10(22.7%)	

A Chi – Square Test. *P*-value of 0.05 was considered significant

## Discussion

This study assessed douching practices among women in the Bolgatanga Municipality of the Upper East Region of Ghana. The majority of the participants of the study douched. Many of them had no knowledge of the dangers associated with vaginal douching. Cleansing of the vaginal was the main reason for douching.

The study found that 66.5% of the women who participated practiced douching. This is lower than what has been reported by other studies investigating douching practices. Ekpenyong et al., reported a prevalence of 80% among young adult women in Nigeria. Furthermore, our prevalence rate is lower than the 84% reported among sex workers in Yunnam Province in China [21]; the 72% among women attending reproductive health clinic in Dar es Salaam in Tazania [19]. However, the reported prevalence in this study is higher than the 46% reported among black women at risk in Southern United States [6].

In this study, over half (67.0%) of the women who practiced douching did so daily. In other studies less than 30% of women who douche did so daily [1, 22]. The high number of women in this study who douched daily is a source of concern for health care services due to the negative health effects of vaginal douching such as bacterial vaginosis, pelvic inflammatory diseases, cervical cancer among others.

Consistent with other studies water was the most common product used (67.7%) [9, 20, 23]. While water may not be seen as a dangerous product for douching, contaminated water may be a source of infection to these women(10,11,). Giving the setting of the study area, the safety of the water used by these women cannot be guaranteed. The women also douched for various reasons which were mainly for vaginal hygiene and vaginal tightening for sexual satisfactions by their spouses. This is consistent with other studies elsewhere [1, 22, 23].

Findings from other studies [1, 22, 23] indicate that mothers, seniors and self-experimentation have been the main sources of information for those that engaged in douching practices. However, in this study, friends (62.4%) and parents (mothers) (17.3%) were core sources of information for participants that have heard and practiced douching with self-experimentation being the least accounting for only 9.0%. This disparity may be due to the cultural differences in the study settings.

Another important finding of the study was that 87.2% of the women who practiced douching, were not aware of its harmful health effects. This is consistent with previous findings [8, 13, 23–27]. In this study, we found that education was associated with awareness of the dangers of douching as well as its practice. Women who had low level of education had inadequate knowledge regarding the dangers of douching and were more likely to practice douching. These findings are consistent with those of previous reports from Nigeria and Uganda [28, 29]. Education is a strong predictor because, educated women have access to health information and are more likely to understand compared to their less educated counterparts.

## Conclusion

Vaginal douching is commonly practiced by women in the Bolgatanga Municipality. Most of these women douche because they did not know that there are health problems associated with the practice. Health education on the issues of women health is very vital for the improvement of women’s health.

## Abbreviation

VD: Vagina douching
